# Fertility-Sparing Treatment for Young Patients with Early-Stage Cervical Cancer: A Dawn of a New Era

**DOI:** 10.3389/fsurg.2022.867993

**Published:** 2022-05-06

**Authors:** Charalampos Theofanakis, Aristotelis-Marios Koulakmanidis, Anastasia Prodromidou, Dimitrios Haidopoulos, Alexandros Rodolakis, Nikolaos Thomakos

**Affiliations:** ^1^ School of Health Sciences, National and Kapodistrian University of Athens, Athens, Greece; ^2^ Division of Gynaecologic Oncology, 1st Department of Obstetrics and Gynaecology, Alexandra Hospital, Athens, Greece

**Keywords:** cervical cancer, fertility preservation, fertility-sparing surgery, radical trachelectomy, less radical surgery

## Introduction

In the last decades, the incidence and mortality rate of cervical cancer in high-income countries have decreased due to the implementation of organized screening programs and recent advancements in diagnosis and prognosis (1). Potential candidates for surgical resection are women with locoregional tumors. Given the excellent 5-year survival rates for early-stage cervical cancer, surpassing 90%, and that up to 40% of these patients are of reproductive age, the need for fertility-sparing surgery (FSS) is mandatory (2). In the literature, many approaches are described, but in the last decade, the debate has been focused on radical and more conservative surgical approaches.

### Vaginal Radical Trachelectomy

Vaginal radical trachelectomy (VRT), combined with laparoscopic pelvic lymphadenectomy, was introduced by Professor Daniel Dargent, back in 1987. The procedure begins with the incision of the vagina and the dissection of the bladder and ureters away from the cervix. Identification and, usually, preservation of the uterine arteries is succeeded through the dissection of the Douglas pouch and the resection of proximal parametria, bilaterally. The minimum tissue needed to be preserved from the uterine isthmus is between 0.5 and 1 cm, and the resection of the specimen includes approximately 1–2 cm of the vagina (3, 4). The specimen is then sent for a frozen section to ensure negative margins, with 5–10 mm being the recommendation in the literature (5, 6). Also, subsequent curettage of the fundus has been reported for the exclusion of remaining residual disease, especially in adenocarcinomas. In case that negative proximal margin in conjunction with the preservation of adequate cervical tissue (5–10 mm) cannot be obtained, radical hysterectomy is recommended (3, 7).

### Abdominal Radical Trachelectomy

Abdominal radical trachelectomy (ART) was introduced by Smith et al. in 1997. The approach to the abdomen is succeeded through a low transverse or vertical abdominal incision, and it is similar to the surgical approach for abdominal radical hysterectomy. Uterine arteries can be preserved or ligated due to the ovarian vasculature that preserves the uterine blood supply.

ART is the standard approach for patients with stage IB1 tumors, as stated by the ESGO/ESTRO/ESP Guidelines (8). However, the main disadvantages include higher blood loss, increased transfusion rates, greater incidence of wound infections, and prolonged hospital stay. The radicality of the parametrial resection in ART is more extensive, compared to the vaginal approach, and the oncologic outcomes are comparable to those after radical hysterectomy, with a recurrence rate of approximately 3.9% (9).

### Minimal Invasive Surgery-Radical Trachelectomy

Minimal invasive surgery-radical trachelectomy (MIS-RT) includes either robotic or laparoscopic radical trachelectomy (RRT or LRT, respectively), and its main target is to accomplish radical parametrial resection as ART without the adverse effects of open surgery. The advantages of the MIS-RT approach are decreased blood loss, lower rates of wound dehiscence, and shorter hospital stay without significant prolongation in operative time compared to ART (10, 11).

The oncologic outcomes of MIS-RT are similar to those of VRT and ART, with a combined recurrence rate of 4.2% and a death rate of <1%; however, the data remain unclear, especially regarding the lesion size, due to the small size of samples in MIS-RT studies (12). However, after the publication of the LACC trial, the minimal invasive approach for young patients with cervical cancer is not recommended, and each treatment should be tailored (10, 13).

### Less Radical Fertility-Sparing Surgery in Early-Stage Cervical Cancer

Although the gold standard for early-stage cervical cancer is radical hysterectomy, related complications regarding principally to the parametrial resection lead to decreased quality of life. The main negative effects include sexual dysfunction, bladder and rectal dysfunction, and fistula formation (14, 15). Furthermore, the rates of parametrial spread have been observed to be <1% in a selected group of patients with negative pelvic lymph nodes, tumor stage IB1, and a depth of invasion of <10 mm (16). The combination of increased possibility for severe complications with findings that show no significant improvement in oncologic outcomes from radical procedures has led to a more conservative surgical approach for early-stage disease. The less radical surgical management includes cervical conization and simple trachelectomy with sentinel lymph node mapping or complete pelvic lymphadenectomy.

Cervical conization refers to the excision of a cylindrical wedge or a cone-shaped resection of the uterine cervix, including the transformation zone. The NCCN guidelines recommend cone biopsy with negative margins in stage IA1 with negative LVSI; however, for stage IA1 with positive LVSI or stage IA2, the recommendations are cone biopsy or radical trachelectomy with pelvic node dissection or the consideration of sentinel lymph node mapping. The management of St IA1 with cone biopsy has been found to have no difference in the 5-year survival rate compared to hysterectomy (17). Simple trachelectomy is defined as the removal of the cervix, leaving the adjacent paracervical tissues *in situ*. It should be combined with lymph node dissection or sentinel lymph node mapping, and it serves as an alternative option for lesions <2 cm and negative pelvic lymph nodes (18).

### Neoadjuvant Chemotherapy and Conservative Management of Early-Stage Cervical Cancer

Neoadjuvant chemotherapy (NACT) poses an alternative approach for bulky tumors larger than 2 cm (19, 20). The philosophy of NACT is to reduce the tumor volume, which could lead to an FSS. There are little data in the literature showing the effectiveness of NACT followed by radical hysterectomy in reducing the size of the lesion in cervical cancer. The responses to NACT can be defined using the pathological outcomes on the trachelectomy\cone specimen as complete response, optimal partial response in cases where residual disease is less than 3 mm, and suboptimal partial response for those with residual disease greater than 3 mm. Globally, the response rate to NACT is reported to be approximately 70%, but the implementation of NACT includes some vague and confusing issues (21). Although NACT can potentially convert a positive node to a negative and proceed with the FSS option, outcomes from studies show a higher recurrence rate and identify node positivity as a negative prognostic factor for FSS (22).

### Postsurgical Follow-Up

Patients after any kind of FSS are arbitrarily counseled for a postponement of pregnancy for 6–12 months to allow the detection of possible recurrent or persistent disease (8). Follow-up visits consist of pelvic examination, Pap smear, and colposcopy, combined with pelvic imaging through transvaginal sonography or MRI. The intervals of surveillance are intended to be every 3–6 months for the first 2–3 years and thereafter every 6–12 months for >2–4 years after surgery due to late recurrences (12). Finally, clearance hysterectomy is not recommended after the completion of childbearing (8).

## Discussion

Since 2014, the NCCN has recommended radical trachelectomy as a fertility-sparing treatment for young women with early-stage cervical cancer. Eligible patients for such surgical procedures are women younger than 40 years of age, with stage IA1 with LVSI, IA2, IB1 with lesions <2 cm, and some selected FIGO stage IB2 patients. Also, as an indicator for FSS, selection should be considered the infertility history and the adequate evaluation by a fertility specialist (23, 24). This workup can be completed within 8 weeks from the diagnosis, without impacting survival (25). Negative factors for FSS are bulky tumors (<4 cm) and lesions with high-risk histologic types, such as neuroendocrine tumors and gastric-type adenocarcinomas, due to the highest relapse rates. However, few data are currently available in the literature on bulky St IB2 tumors and the safety of FSS. Therefore, tailored treatment is necessary for these patients (6, 26).

Preoperative evaluation should concern tumor size and depth of invasion. The combination of clinical examination with pelvic magnetic resonance imaging (MRI) is integral, prior to FSS, in order to estimate the tumor size, depth of invasion, and extension of the disease. Also, the addition of computerized tomography (CT) with or without the use of positron emission tomography (PET/CT) can help to evaluate lymphadenopathy and distant metastasis (2, 3). In some cases, cervical conization is used in addition to punch biopsies because it offers a more valuable and accurate estimation of histology, lymphovascular space invasion (LVSI), and tumor size (27).

Regarding the surgical approach, the open abdominal incision can be avoided by VRT, leading to lower complication rates. On the other hand, there is a need for specialized surgical skills in vaginal radical procedures, which are not universally common. Moreover, the use of VRT in tumors greater than 2 cm is limited due to the compromised oncologic outcomes and increased complication rates (18, 28).

The oncologic outcomes of VRT for early-stage cervical cancer in recurrences, 5-year recurrence-free survival, and 5-year overall survival are comparable to that of radical hysterectomy, with optimal prognosis, while death from the disease is 1.7% (12). The reported combined pregnancy rate is 49.4%, and complications from the procedure itself, such as cervical stenosis or female sexual dysfunction, may lead to reduced fertilization (29). For these reasons, preoperative counseling is necessary. Pregnancies after VRT are considered high-risk for preterm delivery and preterm premature rupture of membranes. Therefore, these women should be advised for extensive prenatal monitoring and planned delivery through a cesarean section. The live birth rate in these pregnancies is approximately 65% (12).

When referring to the abdominal approach, multiple studies have shown a greater recurrence rate for lesions >2 cm with ART, but death from disease is reported at <2% (9, 12). The pregnancy rate ranges between 13% and 67%, the combined live birth rate is 44%, and the severe preterm delivery occurs in up to 50% of the total pregnancies, establishing them as high-risk pregnancies (9).

Patients that are candidates for the laparoscopic or robotic approach should be counseled about the advantages compared with open surgery and for the possible higher recurrence risk and the unclear data regarding the MIS-RT approach. Patients with tumors >2 cm are not candidates for the MIS-RT FSS approach. The combined pregnancy rate is 36.2% and the live birth rate is 57.1%, which are comparable to those for radical procedures (9).

Furthermore, regarding NACT, a phase II multicentric study demonstrated that weekly topotecan, with cisplatin, is effective with acceptable toxicity (30). After the implementation of NACT in patients with complete or optimal partial response, large conization or simple trachelectomy seems to be an adequate option since the probability of occult parametrial disease is very low, while in suboptimal chemo responders, radical surgery is mandatory (21).

Patients should be informed about the possible adverse effects of chemotherapy agents on ovarian reserve and the possible premature menopause. However, favorable obstetrical outcomes that have resulted from NACT are related to fewer gonadotoxic agents and shorter chemotherapy cycles (19).

The hot topic of current treatment is, however, less radical surgery. Studies have shown that the risk of recurrence after conization or simple trachelectomy with lymph node sampling is approximately 4.2%, and the significance of lymph node evaluation is essential for the maintenance of oncologic safety after the nonradical fertility-sparing management. The pregnancy outcomes are superior to radical management, with a pregnancy rate of 55.1% and a live birth rate of 71% (12, 31).

Results of the ConCerv trial demonstrated that less radical surgery with conization and simple hysterectomy is a feasible approach for patients with early-stage cervical cancer. The positive lymph nodes rate was 5%, and the rate of residual disease in the hysterectomy group, following conization, was 2.5% (32). Provided that the ongoing SHAPE and GOG-278 prospective trials will present similar results to the ConCerv trial, we will have a more thorough opinion regarding the potential change in the standard of care for early-stage cervical cancer.

To conclude, fertility-sparing treatment for young patients with early-stage cervical cancer demands a tailored approach that is ever-evolving. New trends lead to less radical surgery, targeting optimizing the quality of life without compromising oncologic safety. If the results of the much-anticipated SHAPE and GOG-278 clinical trials match those of the ConCerv trial, then perhaps we will witness a paradigm shift to a more conservative surgical approach for these patients.
